# *Rheinheimera sp*. T2C2 Bacterial Biofilm for Bioremediation of Cobalt(II)

**DOI:** 10.1021/acsapm.6c00304

**Published:** 2026-05-01

**Authors:** Ellen W. van Wijngaarden, Miranda P. Brunette, Alexandra G. Goetsch, Ilana L. Brito, David M. Hershey, Meredith N. Silberstein

**Affiliations:** † Sibley School of Mechanical and Aerospace Engineering, 5922 Cornell University, Ithaca, New York 14853, United States; ‡ Department of Material Science and Engineering, Cornell University, Ithaca, New York 14853, United States; § Department of Bacteriology, 5228 University of Wisconsin-Madison, Madison, Wisconsin 53706, United States; ∥ Meinig School of Biomedical Engineering, Cornell University, Ithaca, New York 14853, United States; ⊥ Engineered Living Materials Institute, Cornell University, Ithaca, New York 14853, United States

**Keywords:** biofilm, bioremediation, heavy metals, biopolymers, engineered living material, *Rheinheimera*, metal recovery

## Abstract

Toxic metals, including cobalt, are often the cause of the contamination of rivers and lakes in mining regions. Heavy metal water pollution has been linked to numerous human health problems, prompting the need for environmental remediation. Existing techniques for removing heavy metals from water, such as chemical precipitation and filtration, produce toxic waste, are costly, or require high power consumption for pumping. Biosorption is a potential alternative strategy that is cost-effective and uses readily available and naturally produced biomass and living material to absorb pollutants. Engineering living materials, such as biofilms, which consist of living cells and a secreted polymer matrix, offer the potential to integrate toxin sensing, sequestration, and metabolism capabilities of cells to improve pollution remediation strategies. Alternative biofilm producing candidates need to be explored to implement these material capabilities. Previous biosorption studies have primarily used bacterial biofilms from known pathogens and/or generated toxic waste in the form of the absorbent material combined with the heavy metal. Here, we describe a recently isolated bacterium called *Rheinheimera sp.* T2C2 that forms biofilms with promising biosorption characteristics. T2C2 is an aquatic bacterium with low nutrient requirements and high biofilm production that is not known to be pathogenic. We demonstrate (1) the efficacy of *Rheinheimera sp.* T2C2 as a biosorbent for cobalt bioremediation; (2) how biosorption is altered by water conditions to establish the efficacy of this strategy in different environments; and (3) how the metal can be released from the biofilm for metal recycling. Our findings will provide a living materials strategy that overcomes the existing barriers for bioremediation and improves the health of ecosystems and humans through heavy metal removal and recycling.

## Graphical abstract

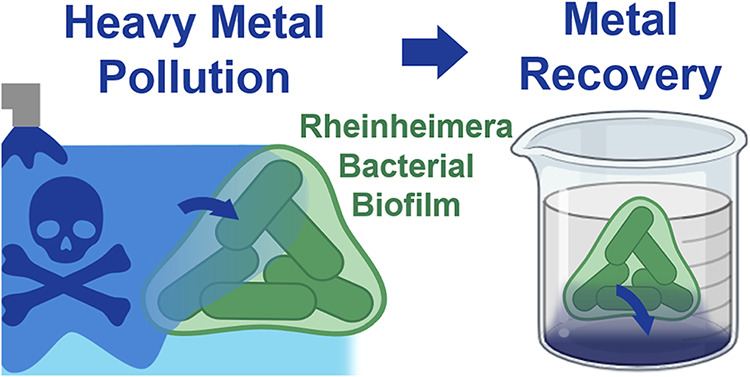

## 1 Introduction

The widespread use of electric vehicles and electronics in daily life has dramatically increased the demand for heavy metal mining. Mining production of cobalt, a key metal for electronics, was over 2.3 × 10^5^ t in 2023, and above 3 × 10^5^ t in 2024.^1^ The European Union estimates that only around 32% of cobalt is recycled, driving the high demand for increased mining, which it turn leads to heavy metal pollution and negative health effects.^2^ Numerous studies demonstrate the toxic effects of heavy metal contamination, including respiratory issues, pancreatic damage, and harm to reproductive organs due to heavy metal ingestion from sources, such as drinking water.^3–6^ The World Health Organization regulates cobalt levels in drinking water to 50 μg/L, although the organization notes that no safe level of cobalt exists.^7^ Cobalt concentrations of up to 34.4 g/L have been measured in mine water, and concentrations from 0.5 to 2028 μg/L have been routinely measured in surface water in Canada, with values likely to increase further due to mining trends.^8^

The increased demand for cobalt mining and the negative health effects due to heavy metal pollution necessitate remediation strategies; however, many of these techniques have drawbacks. Chemical precipitation is simple and cost-effective but produces toxic waste and only works in a narrow pH range.^5,9^ Ion exchange successfully recovers metal at low concentrations in the parts per billion range.^5,9–11^ However, ion exchange is an expensive method for high volumes of effluent, particularly at high metal concentrations, which quickly saturate metal binding sites. Physical methods, such as filtration, are costly, require regular antifouling maintenance, or need high power consumption for pumping.^5,12^ Ultimately, the drawbacks of these chemical and physical strategies for remediation hinder implementation and widespread use.

Bioremediation techniques have gained interest as a means of overcoming some of the disadvantages of current remediation methods. Biosorption presents a cost-effective strategy with minimal toxic waste due to the use of natural, readily available, and often biodegradable materials. Bioderived and engineered living materials (ELMs) present exciting possibilities for enhancing biosorption, to include toxin sensing^13–16^ and sequestration.^5,17^ Current bioremediation techniques use biomass from an array of sources, including plant matter, mushrooms, algae, or biofilms.^18–28^ While most of these examples are primarily academic demonstrations, algae have been deployed commercially for wastewater remediation technologies.^29^ Biosorption using algae packed in columns has proven to be effective; however, this method still requires the disposal of algae or biosorbent with absorbed metal, and synthetic biology tools for working with algae remain limited. Therefore, routes to improve the system from a biological engineering end are limited.^18^ Bacterial biofilms have also proven to be quite effective for metal uptake.^30^ These biofilms are a combination of live cells and self-produced extracellular polymeric matrix, typically rich in high-molecular-weight (MW) polysaccharides, proteins, lipids, and DNA.^31^ Synthetic biology along with tuning environmental factors, such as growth conditions, offer multiple strategies to improve biofilm yield, metal uptake, and biosafety, thereby supporting system level engineering.

Widespread implementation of bacterial biofilm-based ELMs for metal recovery requires wild-type, native, nonpathogenic bacteria that produce high amounts of biofilm and achieve effective metal removal. Previous work has focused on bacteria that meet only some of these requirements, including prolific biofilm producers, such as *Pseudomonas aeruginosa*.^5,32^ Although *P. aeruginosa* is pathogenic, which poses a barrier to widespread use, it has been proven to be effective for heavy metal uptake.^5,32^ Previous work has demonstrated that bacteria of the genus *Geobacter* can take up cobalt and down-cycle it. While this does not allow for metal recovery to decrease the demand for mining, this strategy might be preferable in environments where recovery is not possible or contamination is exceptionally widespread.^28^ The use of wild-type, nonpathogenic bacteria, such as soil strains, for heavy metal uptake holds great potential for widespread use and has been demonstrated in a contained environment with industrial level waste concentrations. This direction is promising but requires additional study in varied environments and lower pollution level concentrations.^33^ We have taken inspiration from this previous work to explore the properties of an aquatic bacterium, which is ideal for water remediation.

An ELM for bioremediation, specifically for cobalt pollution, requires a non- pathogenic, aquatic organism with stable growth and consistent rheological properties across temperature and pH ranges for water environments, such as lake water where cobalt pollution is high.^8^ Additionally, high biofilm production under low-nutrient conditions is needed to maintain the material. Differences in the composition and structure of the biofilm matrix may lead to fundamental differences in bioremediation performance between organisms. For example, the abundance of hydroxyl and carboxyl functional groups, which is a compositional characteristic, would be ideal for metal adsorption. Such an organism would greatly facilitate further design and optimization for metal uptake based on the environmental conditions and exposure time. Here, we describe a newly isolated^34^ bacterium, called *Rheinheimera sp.* T2C2, that produces a biofilm with promising properties for bioremediation of cobalt. We demonstrate (1) the efficacy of *Rheinheimera sp.* T2C2 biofilm as a living material for cobalt bioremediation; (2) how biosorption is altered by water conditions and exposure time to establish the efficacy of this strategy in different environments; and (3) an approach for releasing metal from the biofilm for metal recycling.

## 2 Experimental Section

### 2.1 *Rheinheimera sp.* T2C2 Isolation

A volume of 1 mL of water from Lake Mendota in Madison, WI, was inoculated into a 10% strength PYE medium and allowed to incubate at room temperature without shaking. After 2 days, biomass was extracted from the air–liquid interface of the culture, homogenized, serially diluted, and plated on the PYE agar. A single, mucoid colony was purified by repeated restreaking on PYE agar. The 16S rDNA gene was amplified using PCR and sequenced using the Sanger method to identify the isolate as belonging to the Rheinheimera clade. A large collection of Rheinheimera strains isolated from Lake Mendota will be described elsewhere.^34^

### 2.2 Bacterial Culturing

All reagents were sourced from Sigma-Aldrich (St. Louis, MO) unless otherwise noted. Peptone Yeast Extract (PYE) media, containing 0.2% w/v peptone, 0.1% w/v yeast extract, and 3% sucrose, was used to culture bacteria. A 10 mL starter culture was inoculated with LM7 cells from a frozen glycerol stock and grown at 30 °C overnight on a shaker at 200 rpm. A 10 mL starter culture was added to 1 L of PYE and 3% (w/v) sucrose solution. The culture was grown until approximately the end of day 4 at 18 °C with shaking at 200 rpm. All cultures were grown until an optical density of 0.5 at 600 nm was reached.

### 2.3 Yield Measurements and Optical Density

Optical density of the cultures at 600 nm was measured using a plate reader (Spectroquant, Darmstadt, Germany). The yield of the material was determined by lyophilizing three 10 mL volumes of culture with a known optical density. Samples were left in the lyophilizer (FreeZone 2.5 L Benchtop Freeze-dryer, Labconco, Kansas City, MO) for 24 h until they were fully dry, before they were weighed to determine the yield per volume of culture.

### 2.4 Metal Uptake Tests

After 4 days of growth following the bacterial culturing procedure described above, the culture optical density was adjusted to 0.5 by adding additional PYE medium as needed. The culture was then separated into 5 mL aliquots in 15 mL tubes and centrifuged at 2 × 10^3^ rpm for 5 min to obtain biofilm pellets. The supernatant was decanted, and the pellets were resuspended with 1 mL of deionized water to wash the biofilm to eliminate salts from the media. The resuspended biofilm was centrifuged for a second time at 2 × 10^3^ rpm for 5 min. The 1 mL biofilm pellet was then transferred to an upper insert of a transwell containing the desired cobalt concentration in the lower part of the well. The material was left at the desired temperature and pH for the set exposure time before the upper transwell insert was removed and the remaining cobalt concentration in the free solution was measured. 12-well transwell plates with a polycarbonate 0.45 μm membrane size (VWR) were used for all tests. For pH experiments, sodium dihydrogen phosphate anhydrous/phosphoric acid buffers at pH values of 2 and 9 were used to tune the pH to the desired values. Following metal exposure, 50 μL of biofilm from the upper well was used to make serial dilutions and bead-plated on LB agar plates to measure the viability of cells following metal exposure.

### 2.5 Cobalt Measurement

The cobalt concentration remaining in the free solution following biofilm exposure was determined by using a colorimetric reaction. A sample volume of 600 μL was added to a 50 mL beaker along with 3 mL of a 40% weight/volume sodium citrate in water solution. An indicator solution made up of 0.1% bromothymol blue in 50% ethanol was prepared in advance. A volume of 0.3 mL of indicator solution was then added to the reaction. The solution was neutralized with 10% sodium hydroxide solution added dropwise until the color matched a standard solution (20 mL of water, 5 mL of 40% sodium citrate solution, and 0.5 mL of indicator solution). A volume of 6 mL of 0.5% w/v nitroso-R-salt in deionized water was then added. The solution was brought to a boil, and 3 mL of 34% nitric acid was added. Following 1 min of boiling, the solution was cooled to room temperature and a 200 μL aliquot was transferred to a 96-well plate for measurement on a plate reader at 500 nm. The absorbance of a sample blank without cobalt was subtracted from the sample absorbance value before comparing the sample absorbance against that of cobalt standard solutions to determine the concentration of cobalt.

### 2.6 ζ-Potential and Molecular Weight Measurements

ζ-potential and *M*_*W*_ were measured using a Malvern Nano Zs Zetasizer (Malvern Pananalytical, Malvern, UK) following the protocol used previously.^35^ Solutions were given 2 h to equilibrate before measurement in a polystyrene or ζ-potential folded capillary cuvette for the measurement of either *M*_*W*_ or ζ-potential, respectively. ζ-potential was measured at a concentration of 0.1% with deionized water as the buffer. *M*_*W*_ was determined using the light scattering measurements from five concentrations: 0, 0.025, 0.05, 0.075, and 0.1%. The Rayleigh equation was then applied to calculate the *M*_*W*_ of the sample.^36^

### 2.7 Fourier Transform Infrared Spectroscopy

Fourier Transform Infrared Spectroscopy (FTIR) measurements were conducted using a Bruker Vertex FTIR spectrometer (Bruker Corp., Billerica, MA). A spectral range of 600 to 3500 cm^–1^ was selected. Approximately 5 mg of sample were loaded into the sample compartment to completely cover the attenuated total reflectance (ATR) crystal. A nitrogen atmosphere was used for all reference and sample measurements.

### 2.8 Rheometry

Rheometry testing was performed using a TA Instruments DHR3 rheometer (Texas Instruments, Dallas, TA). A strain sweep at a frequency of 1 rad s^–1^ was used to establish the range of the linear viscoelastic region. A strain of 1% was selected to be used for frequency sweeps based on the results of the strain sweeps. A sweep of strain and frequency was completed for every sample. A 20 mm parallel plate fixture was used with the gap set to 300 μm, requiring a sample volume of approximately 100 μL. Biofilm samples were exposed to desired temperature or pH conditions for 24 h in a transwell plate before being harvested for rheology. The biofilm concentration was 2 mg/mL (dry biofilm weight per mL of water). The parallel plates were thoroughly cleaned before use and between samples with deionized water and isopropyl alcohol and dried with compressed air. The sample was added onto the lower plate, and the top fixture was lowered to the set gap value. Graphs presented are the average of three independent trials.

### 2.9 Alcian Blue Staining

#### 2.9.1 Collection of Floating Biofilms

Overnight liquid cultures were diluted 100-fold into 250 mL glass beakers containing 200 mL of the PYE medium. Cultures were grown statically at room temperature for 5 days to allow for biofilm maturation. Four layers of cheesecloth were secured over the opening of a 1 L glass beaker with a rubber band, and biofilm cultures were carefully poured over the cheesecloth to collect the biofilm matrix. The gelatinous filtrate was then collected with a metal spatula and transferred to a 15 mL conical tube.

#### 2.9.2 SDS-PAGE Analysis of Biofilm Composition

4× Laemmli buffer (250 mM Tris-HCl pH 8.0, 8% w/v SDS, 40% v/v glycerol, 0.05% w/v Bromophenol Blue and 5% v/v β-mercaptoethanol) was added to biofilm extracts to achieve a final concentration of 1×. Solubilized extracts were serially diluted, heated at 95 °C for 5 min, and loaded onto 4–20% polyacrylamide gradient gels (BioRad). After resolving, gels were fixed in a solution of acetic acid/ethanol/water (1:5:4) for 1 h and subsequently washed for 1 h in deionized water. The fixing and washing steps were repeated for a second time. Gels were then stained overnight in a 0.2% solution of Alcian blue, 3% v/v acetic acid, and 50 mM MgCl_2_. A solution of 3% (v/v) acetic acid and 50 mM MgCl_2_ was then used to destain the gels.

### 2.10 Cobalt Cell Uptake Test

Cells were separated from the secreted biofilm to compare the amount of cobalt removed from the solution by the biofilm alone and by the cells. Centrifugation of 5 mL of culture at a maximum possible speed of 7830 rpm (Eppendorf, Germany) for 15 min was used to separate out cells from the biofilm. A volume of 1 mL of biofilm was then carefully pipetted off and placed in a separate transwell plate for cobalt exposure. The 100 μL cell pellet was resuspended in 0.9 mL of water and added to a transwell plate well for cobalt exposure. Both the biofilm and the cells underwent 24 h of exposure to water with a cobalt concentration of 2 × 10^3^ μg/L. The concentration of cobalt remaining in the water was then measured following the exposure. One group of exposed cells were also exposed to 5 mg/mL for 6 h to lyse cells. The cobalt concentration in the free solution was measured after the lysozyme exposure. Cells were found to survive lysozyme exposure if not first separated from the biofilm.

### 2.11 Cobalt Recovery Tests via Degrading Protein

Cobalt recovery was conducted using guanidinium iodide and proteinase K to break down metal binding proteins. A concentration of 10% guanidinium iodide (MS140000, Greatcell Solar, Australia) and 0.2% proteinase K (20E0456058, VWR, Radnor, PA) were used for 6 h. The metal uptake with and without the chemical treatments was then measured to compare the effect of degrading proteins on metal uptake and recovery.

### 2.12 Column Uptake Test

Biofilm pellets with a dry weight of 160 mg were produced by centrifuging culture at 2 × 10^3^ rpm for 5 min. The pellets were washed with deionized water before being transferred to a coffee filter that had been prewetted with deionized water. Four volumes of cobalt contaminated water at a concentration of 2 × 10^3^ μg/L were run through the filter with the pellet. Filtration was gravity driven, and each 10 mL volume was collected in a unique 50 mL Falcon tube placed under the filter. The cobalt concentration of each 10 mL volume was then measured. Tests were performed in triplicate. Results were compared to a sample of deionized water run through the biofilm filter as well as a cobalt sample run through just the coffee filter to eliminate variation in absorbance that was due to cell debris or cobalt adsorption of the coffee filter.

### 2.13 Batch Uptake Test

Liter scale uptake of cobalt was demonstrated by exposing cobalt contaminated water to biofilm for 24 h. Tests were conducted in liter-sized glass bottles. The concentration of cobalt tested was 2 × 10^3^ μg/L with 2 mg of biofilm per 1 mL of water (2 g total biofilm used per each liter container). A cobalt stock solution was made from cobalt­(II) chloride salt at a concentration of 40 g/L. The stock solution was then diluted to make liter volumes of cobalt. After exposure, a 50 mL water sample was taken and filtered to remove any biofilm or cell debris before measuring cobalt uptake from the free solution. Proteinase K was then added for a 6 h exposure at a concentration of 2 mg/L to digest proteins involved in metal uptake and recover the cobalt. Following the proteinase K exposure, another 50 mL of water sample was extracted, filtered, and used for cobalt measurement to calculate metal recovery. All tests were performed in triplicate.

### 2.14 Statistical Analysis

Two-tailed Student *t* tests with equal variances were used to determine whether statistically significant differences existed between groups. *P*-value cutoffs of 0.05, 0.01, and 0.001 are indicated by *, **, and ***, respectively. Data are reported as the mean of *n* = 3 samples unless otherwise noted.

## 3 Results and Discussion

### 3.1 *Rheinheimera sp.* T2C2 as an Ideal Candidate for Heavy Metal Bioremediation

Ideal bacteria for use in ELM development would be nonpathogenic, wild-type, aquatic, and able to survive with low nutrients to ensure viability under varied conditions (e.g., temperature, pH, nutrients) of use. Although bacterial biofilms have been shown to have potential for the bioremediation of pollutants, not all bacteria are ideal candidates. *Rheinheimera* is not known to be pathogenic and has even been found to inhibit the growth of pathogenic bacteria.^34,37,38^ *Rheinheimera sp.* T2C2, first isolated from a water sample from Lake Mendota (Madison, WI), demonstrates the qualities of being an ideal candidate for bioremediation.^34^ The genus *Rheinheimera* includes various aquatic bacteria found in both saltwater and freshwater, as well as soil all over the world.^39–43^ These bacteria have low-nutrient requirements, and therefore, are typically grown in PYE media in a lab setting.^42,44^ PYE is considered a low-nutrient medium compared to lysogeny broth (LB) media which is often used to grow bacteria in laboratories. LB includes 5 times the amount of yeast extract (0.5% w/v) and 10 times the amount of peptone (1% w/v), as well as sodium chloride and tryptone. Previous studies have mapped out the phylogeny of various strains and identified a range of ideal growth conditions depending on the strain.^39,42,45^ These include strains that have been shown to grow well between 10 and 40 °C and at a pH between 7 and 8, conditions typical of lake water environments.^40,42^ Reported lake water conditions can range from approximately 9 to 30 °C and pH values from 6 to 8 according to the National Oceanic and Atmospheric Administration.^46,47^ While biofilms could be grown in ideal conditions before transfer to the environment, cell viability and continuous production of biofilm or intracellular transport of metal could be advantageous for metal removal and continuous remediation. We show that *Rheinheimera sp.* T2C2 grows well at both 20 and 30 °C with minimal growth at 7 °C in PYE media (Figures 1a and S1). Extended growth for multiple days slightly improves biofilm yields but results in a lower optical density value, likely due to autolysis (Figure S1a,b).

**Figure 1:**
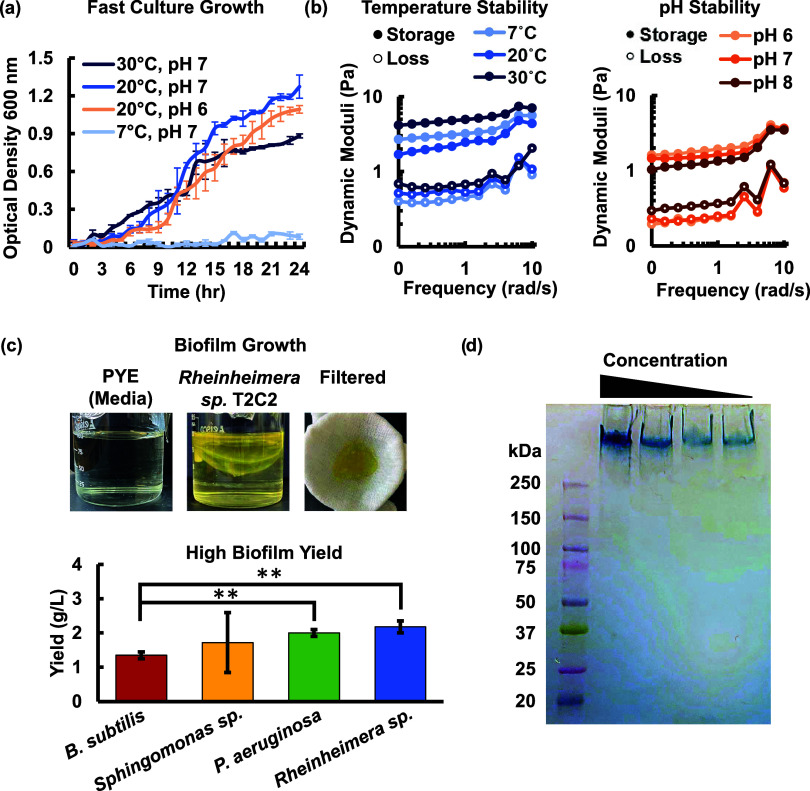
*Rheinheimera sp.* T2C2 biofilm is an ideal biosorbent due to its (a) fast growth in varied environmental conditions with low-nutrient requirements and (b) stable biofilm rheological properties under different environmental temperatures and pH values. (c) The formation of insoluble biofilm with a high yield compared to other model biofilm producing bacteria, such as *Bacillus subtilis*,^49^ *Sphingomonas sp.*,^35^ and *P. aeruginosa*,^48^ would facilitate the use of *Rheinheimera sp.* T2C2 for bioremediation. Mean values are reported and error bars show standard deviation, *n* = 3, **p-value ≤ 0.01 (two-tailed Student *t* test). (d) Alcian blue staining indicates that the biofilm is acidic, providing further evidence for potential for metal binding.

The growth and environmental conditions may also alter the biofilm properties. Rheology was conducted to check the stability of the biofilm under varied environmental conditions. Material stiffness may play a key role in metal diffusivity, and consistent properties are desirable for implementation of a living material. Figures 1b and S2a,b show a stable gel behavior for all lake water-relevant temperatures and pH values with minimal change in the dynamic moduli between conditions. Our results also show growth for pH values of 6 and 7 (Figure 1a). These results indicate that the living material would have consistent properties if applied in various lake water-relevant conditions.

The high biofilm yield of *Rheinheimera sp.* T2C2, compared with other model organisms, is also ideal for bioremediation. T2C2 produces a higher amount of biofilm than *Sphingomonas sp.* LM7, a member of the *Sphingomonas* genus that is often harvested for use as rheological modifiers for use in food, oil and gas, and cosmetics, using the same PYE growth media.^35^ Further, our results show that *Rheinheimera sp.* T2C2 produces a high amount of insoluble biosorbent material, observed as a defined layer of biofilm produced when the bacteria are cultured, rather than a homogeneous solution (Figure 1c). T2C2 produces more biofilm than other high biofilm producers, such as *B. subtilis* (Figure 1c).^48,49^ T2C2 biofilm yield was similar to model biofilm producer *P. aeruginosa* 215. However, unlike *P. aeruginosa*, T2C2 is not known to be pathogenic. It is noted that the examples for *B. subtilis* and *P. aeruginosa* are in their respective preferred media and growth conditions.

The composition and structure of the biofilm produced indicate that this material might be particularly well suited for metal uptake for bioremediation applications. The amount of metal uptake and how the metal interacts with the biofilm would depend on environmental conditions and factors related to the metal, such as oxidation state and electronegativity, in addition to biofilm composition.^50,51^ The biofilm is composed primarily of polysaccharides with additional protein, RNA, DNA, and lipids (Figure S3a). The biofilm is approximately 5% protein by dry weight (Figure S3a) and is mainly composed of high-molecular weight polysaccharides. The purified polysaccharide molecular weight was approximately 11.7 MDa with a surface charge or ζ-potential value of −44.5 ± 2.4 mV. The low ζ-potential value, below −30 mV, indicates that the polysaccharide molecules will repel one another rather than aggregate in solution.^35,52,53^ This is an ideal characteristic for bioremediation to ensure that the biofilm will mix with a desired heavy metal or toxin rather than flocculate out of solution. The acidic nature of the extracellular polymers, as shown in Figure 1d, may also help with metal binding as acidic polymers can donate electrons to metal ions to form stable bonds.^54,55^ The blue color of the Alcian blue staining indicates acidic functional groups, which were further explored by using FTIR (Figure S3b). Key functional groups identified, such as amides, carboxylic acids, and alcohols, may facilitate binding to metal ions (Figure S3b). T2C2 shows strong stretching for amide I for C = O at 1680–1630 cm^–1^, NH at 1580–1515 cm^–1^, alcohol groups for C–O at 1075–1000 cm^–1^, and C = O from 1670 to 1650 cm^–1^, indicative of internal hydrogen bonding. The presence of carboxyls, amines, and hydroxyls indicates potential for metal binding and effectiveness for bioremediation. The combined compositional evidence points toward the potential of the T2C2 biofilm for metal uptake.

### 3.2 Biosorption of Cobalt Using *Rheinheimera sp.* T2C2

Cobalt biosorption was tested to compare T2C2’s performance to other biosorbents. T2C2 biofilm pellets were exposed to water containing varied concentrations of cobalt chloride salt, within an environmentally relevant range, in a transwell plate. The transwell membrane size of 0.45 μm prevented any cells from migrating into the water below the transwell but the metal ions were free to diffuse into the biofilm in the upper part of the transwell (Figure 2a, inset). A colorimetric reaction with the nitroso-R-salt was used to measure the change in cobalt concentration in the free solution following biosorption (Figure S4). Increasing concentrations of cobalt resulted in a lower percentage metal removal as the metal binding capacity of the biofilm was saturated at higher concentrations (Figure 2a). However, the overall amount of metal removed was still higher due to the increase in initial solution concentration.

**Figure 2:**
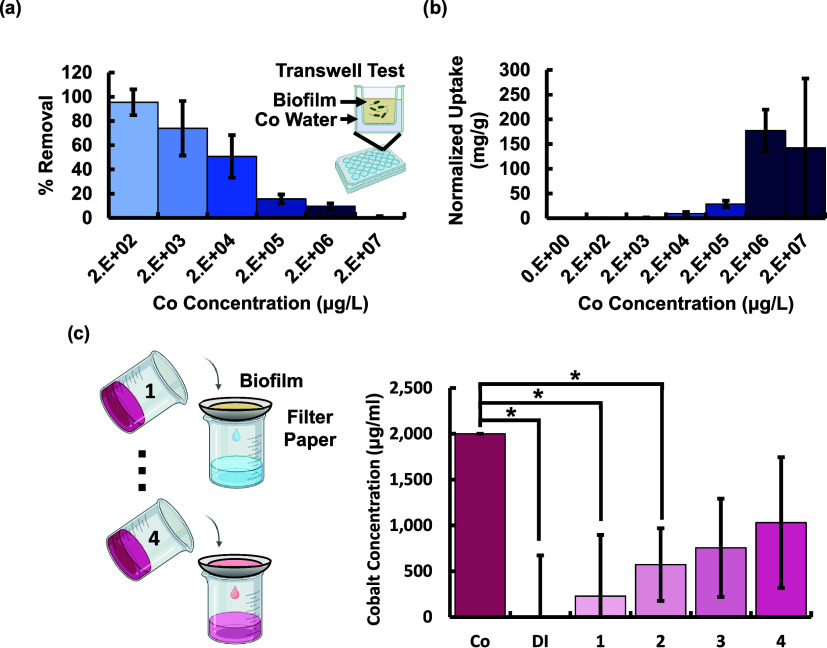
*Rheinheimera sp.* T2C2 demonstrated effective bioremediative properties showing effective (a) removal of cobalt from water and (b) metal uptake normalized by biofilm mass for a range of environmentally relevant metal concentrations spanning 200 μg/L to 20 g/L. (c) Metal could also be removed via filtration using biofilm pellets to achieve comparable uptake to the transwell experiments while decreasing exposure time. Aliquots 1 to 4 of cobalt contaminated water was poured through a coffee filter containing the biofilm pellet in 10 mL increments, and each volume of filtered water was collected individually for cobalt measurement. The initial cobalt concentration was 2 × 10^3^ μg/L. Tests were performed in triplicate, error bars show standard deviation. Mean values are reported, and error bars show standard deviation, *n* = 3, *p-value ≤ 0.05 (two-tailed sSudent *t* test).

The normalized uptake of the biofilm (Figure 2b) was determined to add insights into the effectiveness of biofilms at different cobalt concentrations and to facilitate comparisons with other biofilm producers. Normalized uptake, sometimes termed uptake capacity (*Q*), was calculated by multiplying the difference in the initial (*C*_initial_) and final metal concentrations (*C*_final_) by the volume of cobalt contaminated water (*V*) and dividing by the biofilm mass (*M*):

1 $$Q=\frac{V\left(C_{\mathrm{initial}}-C_{\mathrm{final}}\right)}{M}$$

A peak value of uptake was obtained for 2 × 10^6^ μg/L cobalt, with metal uptake likely saturating above this concentration. At 200 μg/L, the Q-value for T2C2 was 175 μg/g (95% removal) compared to *P. aeruginosa* which took up 1.19 μg/g (25% removal) under similar initial metal and water conditions (pH 7, room temperature, and 24 h of exposure).^5^ Uptake for another high-performing biosorbent reported in the literature, seaweed derived from *Sargassum wightii*, was 16 mg/g for an initial cobalt concentration of 100 mg/L (16% removal) and 12 h exposure. T2C2 showed comparable metal uptake of 13 mg/g but at a lower initial cobalt concentration of 20 mg/L (65% removal) for the same exposure time, indicating better bioremediation capacity.^56^

Uptake can also be achieved with short exposure by running contaminated water through a coffee filter (Melitta, Germany) containing a biofilm pellet, presenting a possible alternative strategy for gravity-driven filtration that does not require long exposure periods. It is noted that using a filter to directly remove ions would require a pore size of approximately 6 nm,^57^ necessitating high pumping energy compared to a 10 μm pore size^58^ for a coffee filter which proved effective at holding the biofilm pellet and allowing water to flow through due to gravity. We demonstrated this option by placing the biofilm pellet on the coffee filter while 10 mL volumes of cobalt contaminated water were incrementally filtered and collected. This technique yielded significant metal uptake for the initial samples collected. Metal percent removal was comparable to the transwell tests due to the high amount of biofilm used. As expected, the percent of metal removal declined for repeated exposure to cobalt contaminated water, indicating a gradual saturation of uptake sites (Figure 2c). Debris from the coarse coffee filter likely contributes to the high error, as this may affect the absorbance values read during cobalt measurement. The first volume filtered allowed only 229 μg/L through the filter or 11% metal remaining, while the fourth volume had a filtered concentration of 1030 μg/L or 52% metal remaining. Our experiment demonstrates that our first filtration cycle yields filtrate that is statistically similar to DI water and statistically different from the cobalt water (*p*-value ≤ 0.05), indicating a high amount of cobalt removal. Over each cycle, more cobalt binding sites are filled due to repeated exposure to cobalt. By the third cycle, the filtrate becomes statistically similar to the initial cobalt water. Reducing the cobalt concentration in the water to below a specified level, such as the EPA cobalt limit, would be possible through either repeated exposure to fresh biofilm or longer exposure time, as done in the transwell plate tests. Based on our percent cobalt removal for the first cycle of our filtration experiment, we would expect a cobalt concentration of 24.2 μg/L, well below the EPA limit of 50 μg/L, if we reran the cobalt through fresh biofilm. It is noted that we also achieved sufficient cobalt uptake to meet the EPA limit by the third cycle in our column experiment when not using fresh biofilm. This demonstrates how bioremediation could be scaled to increase filtrate volume and reduce time given the high biofilm yield and low cost of production.

It is important to note that high cobalt concentrations can be cytotoxic to bacteria, hindering material production in living materials, which is essential for continued metal sorption. We found that cells were capable of growing biofilms in the presence of low concentrations of cobalt. The biofilm had consistent water content (Figure S5a), yield (Figure S5b), and rheological properties (Figure S5c,d) at cobalt concentrations up to 2 × 10^3^ μg/L. Without initial biofilm matrix to protect cells, 2 × 10^4^ μg/L was too high of a cobalt concentration (Figure S5a,b) for cell growth. However, already grown cells within a mature biofilm matrix could withstand higher cobalt concentrations (Figure S6a). We measured cell viability after exposure in the transwell plate for cells with a mature matrix prior to cobalt exposure. Cobalt had minimal impact on cell viability up to a concentration of 2 × 10^4^ μg/L (Figure S6a), at which point there was a dramatic drop off with increased concentration. FTIR-ATR results indicated that the biofilm matrix is important not only for cell viability but also for metal uptake, clearly visible at high concentrations (Figure S6b). Results show changes in the peaks, particularly in the protein region between 1580 and 1515 cm^–1^, indicating metal binding to these groups. Since our results indicate that cells without a mature matrix are susceptible to cobalt exposure, growing the biofilm in advance under ideal conditions before exposure might be a more effective bioremediation strategy than growing biofilm in contaminated water to better protect cells and promote metal uptake.

### 3.3 Effect of Varied Water Temperature and pH on Biofilm Biosorption of Cobalt

Temperature and pH must also be considered when designing a living material for bioremediation. Living organisms may respond adversely to temperature or pH extremes, with environmental conditions likely influencing cell viability and, perhaps, also metal uptake. While temperature and pH are often beyond human control, exposure time could be potentially modulated to achieve effective uptake. We tested a range of environmental conditions to determine how the temperature and pH affect metal uptake. The role of exposure time was then examined in addition to these environmental factors.

Varied water temperature conditions have been shown to affect uptake performance of biofilms.^5^ We therefore examined uptake at three temperatures, 7, 20, and 30 °C, chosen based on the range of temperatures observed in lake water.^59,60^ We first grew biofilm under consistent ideal conditions before exposure to the three temperatures of interest. We observed metal uptake at all conditions tested (Figure 3). For 6 h exposure time, our results show the highest uptake for 20 °C. However, all temperatures eventually reached similar metal percent removal by 24 h (Figures 3b and S7a). At the 24 h time point, temperature did not alter the percent removal (Figures 3 and S7a). Lower metal percent removal was observed for higher concentrations of cobalt, indicating a gradual saturation of the cobalt binding sites (Figure 3a). We observed the fastest cobalt removal at 20 °C with metal concentrations already statistically significantly altered by 6 h for 2 × 10^3^ μg/L cobalt in wells with biofilm compared to wells without biofilm at 20 °C (Figure 3b). All concentrations of cobalt showed statistically significant metal uptake by 12 h at 20 °C. In contrast, samples at 7 °C showed statistically significant uptake by 12 h at a concentration of 2 × 10^3^ μg/L, and samples at 30 °C did not show any statistically significant uptake until 24 h (Figure 3b). Changes in temperature may influence several factors, including metal solubility, bacterial cell wall configuration, ionization energy of metal–biomass complexes, diffusivity, and reaction equilibrium. Metal coordination complexes can be formed endothermically or exothermically. For example, metal cations and carboxylate ligands form a complex endothermically, whereas amide ligand complexes form exothermically.^61,62^ This might obscure clear trends for metal uptake and contribute to a high variation in data. Previous studies have indicated that an increase in temperature can decrease the capacity for physical adsorption, aligning with our observations that 30 °C results in lower metal uptake than 20 °C initially.^63,64^ However, this does not appear to have an effect at longer exposure times. Viability was highest at 20 °C for all concentrations and time points (Figure S7b). The number of colony forming units per milliliter (CFU/ml) gradually decreased with increasing exposure time and increasing cobalt concentration, indicating the need for investigating the role of active cell uptake compared to cell surface or biofilm sorption.

**Figure 3:**
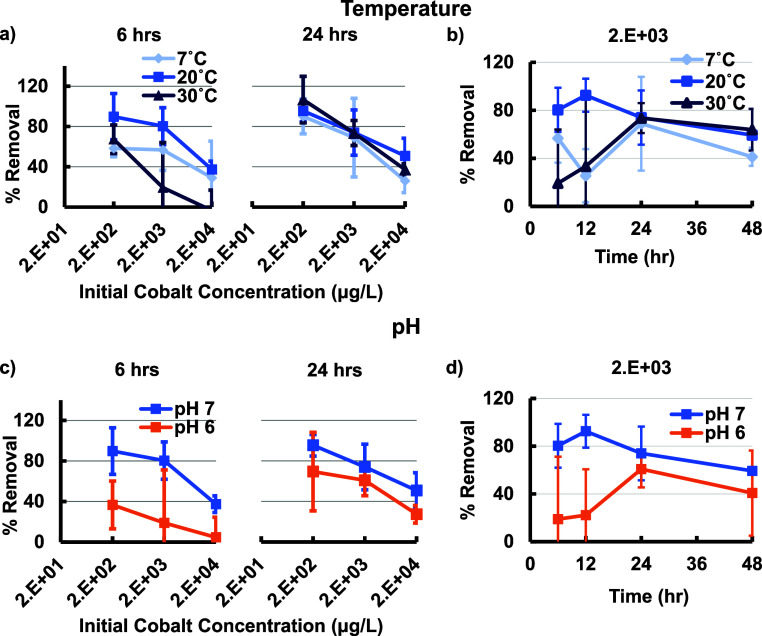
(a) Effect of varying water temperature for exposure times of 6 and 24 h. (b) Varying water temperature showed varied percent cobalt removal peak uptake times. The effects of temperature lessen for longer exposure times. (c) The effect of varying pH for exposure times of 6 and 24 h, on the percent cobalt removal showing a decreasing trend with metal concentration. (d) The effect of varying water pH and exposure time on percent cobalt removal showed differing peak uptake times depending on pH. Mean values are reported, and error bars show standard deviations (*n* = 3).

Varied water pH also affected uptake performance of the *Rheinheimera sp.* T2C2 biofilm grown under standard conditions. The pH of the surrounding water may alter the nature of binding sites and the solubility of metals. Biofilms may contain both weakly acidic and weakly basic groups depending on composition.^65^ A decrease in pH increases the H^+^ ions in the solution, leading to a positive charge on the cell and biofilm surfaces, which could inhibit attraction between metal and biomass.^30,63^ In contrast, an increase in pH has been shown to increase biofilm metal normalized uptake due to the net negative electrostatic surface charge, matching our observations.^63^ For cobalt­(II), the optimal pH, according to the literature, is between 4 and 7, as a pH of approximately 8 and above results in cobalt removal by hydroxide precipitation, creating high volumes of sludge that is difficult to dispose of.^63,66^ This process interferes with cobalt removal by adsorption.^66^ We explored the effect of pH on metal uptake of our living material by testing pH values of 6 and 7 to match the pH of typical lake water.^59^ Previous studies have explored broad pH ranges to establish trends in metal binding and indicated that a pH close to neutral is ideal.^5,18,63^

We observed higher uptake for pH of 7 in comparison with pH 6, except for at a metal concentration of 200 μg/L and an exposure time of 48 h (Figure S8a). A downward trend in percent metal removal with an increased concentration of cobalt was observed for both pH values (Figure 3c). The time for percent removal to equilibrate was higher for a pH of 6 compared to that at a pH of 7, particularly at lower concentrations (Figure 3d). The drop in metal removal for 48 h compared to shorter exposure times may be due to the degradation of biofilm over time.^63^ Viability was higher for a pH of 6 compared to a pH of 7 for all concentrations and exposure times (Figures S8b and S9b), indicating that more cells does not necessarily lead to higher metal removal and that pH can alter multiple factors that are important for bioremediation.

### 3.4 Biofilm Mode of Cobalt Uptake

Establishing the mode of uptake may identify approaches to improve cobalt removal as well as possible cobalt recovery strategies. Metal uptake may occur due to intracellular accumulation, cell surface sorption, and extracellular accumulation. Extracellular vs intracellular uptake is challenging to study as transport of heavy metals into the cell often leads to toxicity and cell death. Cells have mechanisms for uptake of essential ions such as sodium and potassium, which may also lead to the uptake of heavy metals.^5^ Cell surface and extracellular adsorption is likely due to binding with functional groups present on the cell membrane and secreted biofilm, including hydroxyl, carboxyl, and amino groups (Figure S6b).^4,5^ These functional groups contribute to the negative surface charge of the T2C2 biofilm matrix, with a ζ-potential (−44.5 ± 2.4 mV) comparable to other bacterial polysaccharides, such as *Sphingomonas sp.* LM7 with a surface charge of −31.8 ± 2.7 mV and gellan gum with a value of −29.1 ± 3.0 mV.^35,67^ The negative surface charge may assist with the uptake of positively charged cobalt ions. The nitrogen atom from amine groups and the oxygen atom from hydroxyl and carboxyl groups can bond with the cobalt metal ions.^4,5,68^ The lower electronegativity of nitrogen, compared to oxygen, leads to a higher bonding efficiency.^68^ Different cell surface components may also contribute to uptake reactions via various functional groups. Lipopolysaccharide (LPS) plays a role primarily in carboxyl and hydroxyl reactions. Protein is also involved in most cell surface adsorption events as reported for *P. aeruginosa*.^61^ Additionally, phospholipids have also been identified as a key component for hydroxyl-metal binding, demonstrating the vast array of binding mechanisms.^69–71^

We conducted separate metal uptake tests with cells and biofilm to assess the role of active cellular uptake and determine whether extracellular matrix or cells contributed more to metal removal for *Rheinheimera sp.* T2C2. The biofilm was centrifuged to separate the matrix from the cells before metal removal experiments. Results indicate that the matrix portion had statistically significantly more cobalt uptake than the cell component (Figure 4a). It is noted that centrifugation did not completely separate the matrix and cell components as some colonies still grew when the matrix was plated (Figure S10a). Separating the matrix and the cells made the cells much more susceptible to cobalt exposure. Cobalt exposure killed all separated cells, which were not protected by a biofilm matrix and also resulted in significant cell death to cells still embedded in the matrix (Figure S10a). This could be due to the combined stress of centrifuging the biofilm at high speed (7830 rpm) and the metal treatment. It is noted that a higher speed was used here to actually separate cells from film, not merely to collect biofilm from culture solution. The overall number of cells in the matrix and cell components added to approximately the same as the whole biofilm, indicating that centrifuging alone did not significantly affect viability. Separated cells were treated with lysozyme to lyse cells and eliminate active intracellular uptake but preserve cell surface sorption to determine how physiological state, living versus dead, altered cobalt uptake. It is noted that lysozyme was ineffective for lysing cells without prior centrifugation and separation (Figure S10b) as the extracellular matrix protected cells embedded within pores,^72^ resulting in no change in the percent cobalt removed (Figure S10b,c). Separating the matrix component did not significantly alter the FTIR spectra compared with the intact biofilm (Figure S10d). Together, these tests indicate that the intracellular uptake does not play a large role in cobalt removal, supporting that uptake is primarily due to functional groups on the extracellular matrix or cell surface. The cobalt uptake due to the matrix portion was not statistically significantly different from the cobalt removal measured for the biofilm as a whole (Figure 4a).

**Figure 4:**
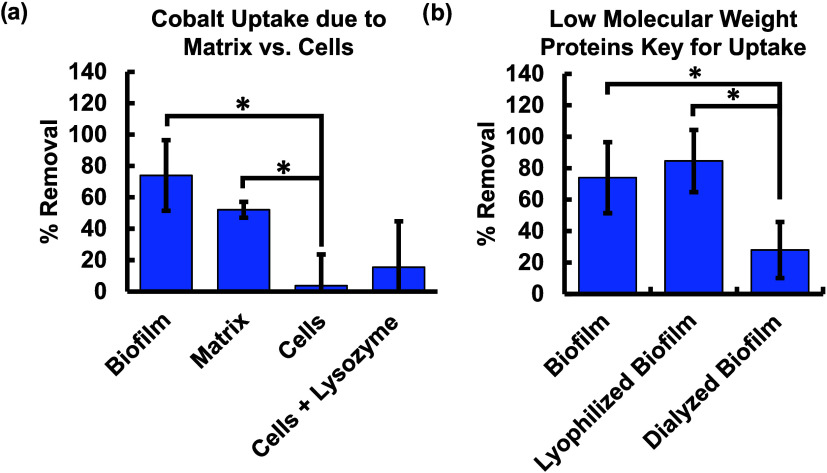
(a) The mode of cobalt uptake (intracellular vs extracellular) was explored by separating and lysing cells from the biofilm before measuring metal uptake, demonstrating that uptake was primarily due to the matrix component. (b) Lyophilization and 100 kDa dialysis post lyophilization were used to kill all cells and eliminate low-molecular-weight proteins, respectively, to determine the component (polysaccharide or proteins) of the matrix that contributed most to cobalt uptake. Mean values are reported, and error bars show standard deviation, *n* = 3, *p-value ≤ 0.05 (two-tailed Student *t* test).

Based on the prior determination that the extracellular matrix was more critical than the active cells for uptake, we set out to determine what component of the extracellular matrix was most important. Specifically, we determined whether matrix polysaccharide or proteins were the primary components for cobalt uptake. To isolate the effect of the matrix metal uptake without living cells, the biofilm was lyophilized, resulting in complete cell death without the addition of any chemicals, such as lysozyme. Dialysis was used, following lyophilization, to eliminate proteins under 100 kDa, to investigate which component of the matrix contributed most to uptake, low-molecular-weight proteins, or the high-molecular-weight polysaccharide. No significant difference was observed between biofilm cobalt removal that was lyophilized and the initial biofilm, indicating that lyophilization did not alter the biofilm structure or composition in a way that would affect cobalt uptake. In contrast, 100 kDa dialysis for 24 h did result in a statistically significant decrease in cobalt removal, indicating that low-molecular-weight proteins play a significant role in cobalt binding (Figures 4b and S3). It is noted that proteins may play a direct role in binding metals or have an indirect effect via altering polysaccharide conformation to affect metal binding.^73^ It is also important to note that small molecules could also play a role, despite the lower concentration or other molecules, such as extracellular DNA (Figure S3a), compared to proteins in the biofilm. This experiment identifies proteins as a key biofilm component for cobalt uptake.

### 3.5 Cobalt Recovery via Biofilm Protein Degradation

Due to the key role of proteins in the cobalt uptake of *Rheinheimera sp.* T2C2, breaking down proteins presents a possible strategy for cobalt recovery for recycling purposes. Biofilms were treated with proteinase K and guanidinium iodide to explore targeting proteins as a strategy for cobalt recovery. These chemicals primarily target the protein component of the biofilm and are more cost-effective than the use of enzymes to break down the polysaccharide component of the biofilm, a strategy that is also limited by specificity and an understanding of the linkages and bonds comprising the polysaccharide structure. Proteinase K is a serine protease used to digest proteins and remove enzymes that break down nucleic acids. It is known as a broad spectrum protease and is capable of breaking down a variety of proteins by bonding to the carboxylic side of both aromatic and aliphatic amino acids to digest and inactivate proteins and enzymes. Guanidinium iodide and other guanidinium salts have been identified as effective protein denaturants.^74^ The literature indicates that guanidinium salts primarily target proteins but may also degrade or alter polysaccharide and other biopolymer structures.^74^ We hypothesized that these methods of digesting and disrupting the protein structure could be used for reversing metal binding for possible recovery.

Our results show significant changes in the biofilm structure and cobalt binding when exposed to proteinase K or guanidinium iodide. Treatment with either chemical significantly reduces the dynamic moduli of the biofilm gel, indicating structural changes to the biofilm (Figures 5a and S11a). Subsequent chemical treatment for biofilm that had taken up cobalt showed that cobalt could be recovered from the biofilms (Figure 5b). Exposure to guanidinium also resulted in 100% cell death, whereas the majority of cells survived proteinase K treatment (Figure S11b). Guanidinium treatment resulted in a statistically significant amount of cobalt recovery in the free solution (Figure S11c). Proteinase K also resulted in the release of metal into the free solution; however, the change was not statistically significant for our initial transwell experiments (Figure S11c). Guanidinium treatment resulted in 100% cell death, whereas the majority of cells survived proteinase K treatment, enabling inoculation of future cultures with the surviving cells (Figure S11b). Due to the toxicity of guanidinium, proteinase K may pose a preferable metal recovery strategy. Proteinase K efficacy is known to improve with temperature, with optimal performance between 20 and 60 °C.^75^ Therefore, conditions could be further altered to optimize the chemical treatment for maximum metal recovery.

**Figure 5:**
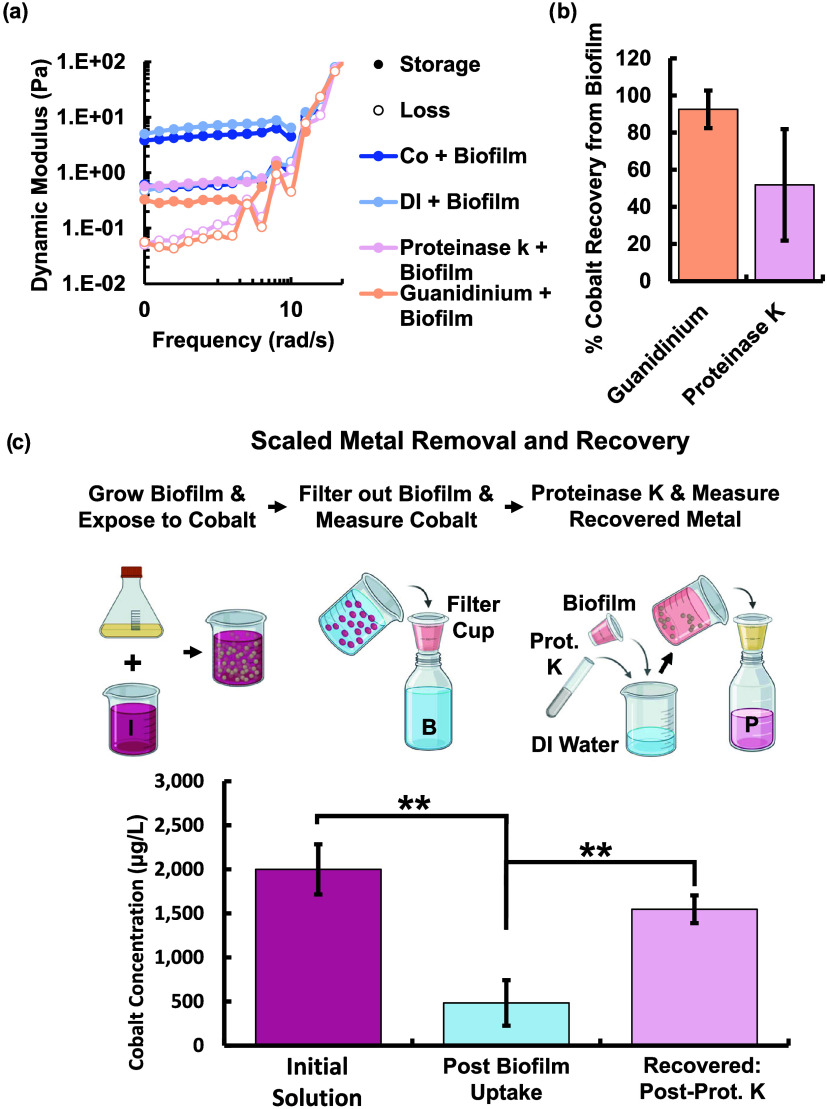
Biofilm was degraded for the purpose of metal recovery using proteinase K (0.2% w/v) and a protein denaturant, guanidinium iodide (10% w/v), for 6 h, respectively. (a) Both chemicals successfully degraded the biofilm, indicated by the decrease in biofilm dynamic moduli, and (b) resulted in return of cobalt into the solution. (c) Schematic of the cobalt uptake and recovery process performed for liter scale water volumes. The biofilm was grown (2 g dry weight), harvested, and exposed to 1 L of cobalt contaminated water at the initial metal concentration. The biofilm was then separated from the free solution to determine the amount of cobalt that was taken up. Finally, the biofilm was treated with proteinase K in a water bath, filtered out, and the recovered cobalt in the water was measured. The cobalt measurements for these three key steps are shown. All graphs show mean and error bars show standard deviations (*n* = 3), **p-value ≤ 0.01 (two-tailed Student *t* test).

Other metals present under environmental conditions may also alter cobalt uptake and potential recovery. We first tested a solution of cobalt (2000 μg/L) with a second metal, iron (1000 μg/L), to determine how cobalt uptake would vary. We were able to measure the concentration of cobalt accurately, as the iron does not interfere with the nitroso-R-salt assay for cobalt detection. The assay step using nitric acid oxidizes out trace metals such as iron.^76^ Post biofilm exposure measurements indicated that the presence of iron did not statistically significantly change cobalt uptake in the T2C2 biofilm (Figure S12a). These tests would indicate that the biofilm has some specificity for cobalt metal uptake. This may be due to cobalt’s ability to bind more readily to some proteins than iron and even replace iron in certain protein complexes.^77^ We also tested a cocktail with metals that are known to have a higher binding affinity than cobalt. The cocktail was made with common metal pollutants including cobalt (2000 μg/L), iron (1000 μg/L), nickel (470 μg/L), manganese (2.2 μg/L), and zinc (120 μg/L). We used values matching metal concentrations in lake water.^78,79^ Multiple metals appear to be taken up by the biofilm, as indicated by the decreased percent cobalt removal for the metal cocktail (Figure S12a). The lack of a statistically significant change is likely due to low metal concentrations.

A scaled cobalt uptake and recovery test was then performed to test the potential for larger-volume metal removal and recycling. A liter of cobalt contaminated water was exposed to biofilm at a concentration of 2 mg/mL. After 24 h of exposure, a sample of the biofilm solution, approximately 100 mL, was extracted and filtered to remove the biofilm. The amount of cobalt removed from the water was measured to infer the amount of cobalt that had been taken up. The biofilm containing cobalt was then treated with proteinase K to release the metal for the measurement of the cobalt recovery. The test demonstrated statistically significant uptake and recovery of cobalt using proteinase K and also showed the potential for liter scale bioremediation (Figure 5). Our current cost due to the use of proteinase K is $0.11 per mg of cobalt recovered.^75^ Although this is significantly more than the raw material cost of cobalt ($0.00006 per mg of cobalt), it is similar to the cost of electrochemically recovering cobalt from solution ($0.08/mg of cobalt).^80^ It is noted that the estimate for electrochemical recovery does not consider the cost of environmental damage due to electrochemical waste. For a better comparison of the proteinase K strategy and other electrochemical recovery methods, one would first need to optimize cobalt recovery using proteinase K, consider the scale of the desired recovery project and the amount of metal needed to be removed, and account for the cost of environmental cleanup due to electrochemical reaction waste from alternative strategies. Additionally, detailed economic viability would depend on the location and regulatory factors in the region. These factors may provide the foundation for an in-depth analysis for specific case studies, which are beyond the scope of this work.^81^

## 4 Conclusion

Biosorption of pollutants and toxins, particularly heavy metals, offers a promising bioremediation strategy that could reduce reliance on conventional electrochemical and filtration-based methods, which can be energy-intensive and costly and generate substantial chemical waste. The design of living materials has the potential to elevate toxin remediation to include sensing, sequestration, and metabolism of targeted species. However, existing biosorbents often rely on pathogenic bacteria to achieve sufficient biofilm production, and few systems enable efficient metal recovery to support recycling efforts and minimize toxic biomass disposal. In this work, we investigated *Rheinheimera sp.* T2C2’s performance for bioremediation applications relative to current biomass remediation strategies. The lack of pathogenicity, wild-type, and aquatic characteristics of T2C2 render the bacterium well suited for biosorption applications. T2C2 exhibits high growth across a range of environmental conditions, possesses matrix functional groups associated with high-affinity metal binding, and achieves a superior biofilm yield relative to commonly studied biosorbents. Our results show that the T2C2 biofilm demonstrates significantly enhanced cobalt uptake under comparable conditions compared to biosorbents such as *P. aeruginosa* biofilm and seaweed, underscoring its potential as an effective and scalable bioremediant. Moreover, we introduce two recovery strategies that enable cobalt release and collection, providing a preliminary path toward integrated biosorption and recovery for metal recycling. Together, these findings position *Rheinheimera sp.* T2C2 as a compelling new candidate for living material-based cobalt recovery for bioremediation. Future use will require additional analysis of the cost based on key factors, such as nutrient component availability for biofilm production, amount of biofilm needed due to metal levels, and bioremediation location. Continued development of ELMs through leveraging such biofilm-forming organisms will be essential to advance sustainable pollution mitigation and improve environmental well-being and human health.

## Supplementary Material



## Notes

Additional experimental details and photos including growth curves, rheological strain sweeps, FTIR-ATR spectra of the biofilm, and all trials for metal removal and viability (PDF)
