# Supplementary material for: Rheinheimera sp. T2C2 Bacterial Biofilm for Bioremediation of Cobalt(II)
Source: ACS Appl Polym Mater. 2026 May 1;8(10):7168–80. doi: 10.1021/acsapm.6c00304 (PMC13200166; doi:10.1021/acsapm.6c00304)
Supplement: Supplementary file 1 [file ap6c00304_si_001.pdf]

Supporting Information: *Rheinheimera sp.* T2C2 Bacterial Biofilm for Bioremediation of Cobalt (II)

Ellen W. van Wijngaarden [‡,⊥], Miranda P. Brunette [¶,⊥], Alexandra G. Goetsch[§], Ilana L. Brito [‖,⊥], David M. Hershey [§], and Meredith N. Silberstein [*,‡,⊥]

‡*Sibley School of Mechanical and Aerospace Engineering, Cornell University, Ithaca, NY 14853, USA*
¶*Department of Material Science and Engineering, Cornell University, Ithaca, NY 14853, USA*
§*Meinig School of Biomedical Engineering, Cornell University, Ithaca, NY 14853, USA*
‖*Department of Bacteriology, University of Wisconsin-Madison, Madison, WI 53706, USA*
⊥*Engineered Living Materials Institute, Cornell University, Ithaca, NY 14853, USA*
∗ *Corresponding Author*

E-mail: meredith.silberstein@cornell.edu
Phone: 607/255-5063

Contents

Supporting Figures

1.0 Ideal Characteristics of *Rheinheimera sp.* T2C2 for Heavy Metal Bioremediation . 2
Figure S1 2
Figure S2 2
Figure S3 3
2.0 Biosorption of Cobalt using *Rheinheimera sp.* T2C2 4
Figure S4 4
Figure S5 5
Figure S6 6
3.0 The Effect of Varied Water Conditions on Biofilm Biosorption of Cobalt 7
Figure S7 7
Figure S8 8
Figure S9 9
4.0 Biofilm Mode of Metal Uptake. 10
Figure S10 10
5.0 Cobalt Recovery via Biofilm Protein Degradation. 11
Figure S11 11
Figure S12 12

Supporting Figures

1.0 Ideal Characteristics of *Rheinheimera sp.* T2C2 for Heavy Metal

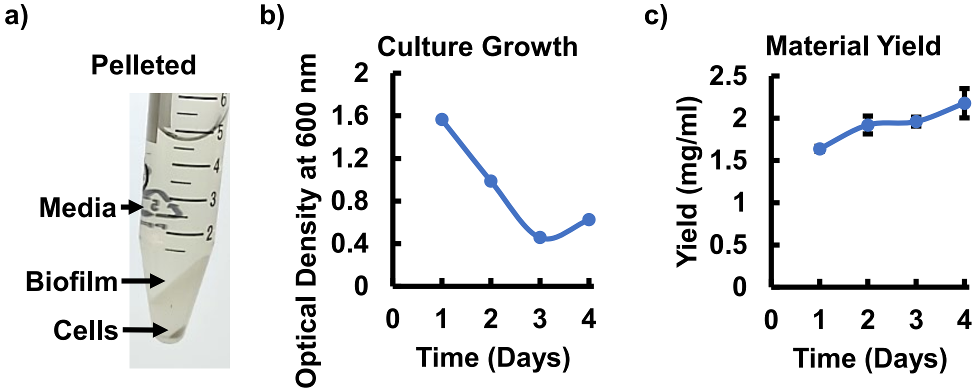

Figure S1: a) The biofilm pellet can be grown and collected via centrifugation of the culture. b) Optical density and c) yield of *Rheinheimera sp.* T2C2 over a four day growth period before biofilm is harvested. Mean values are reported and error bars show standard deviation (n=3).

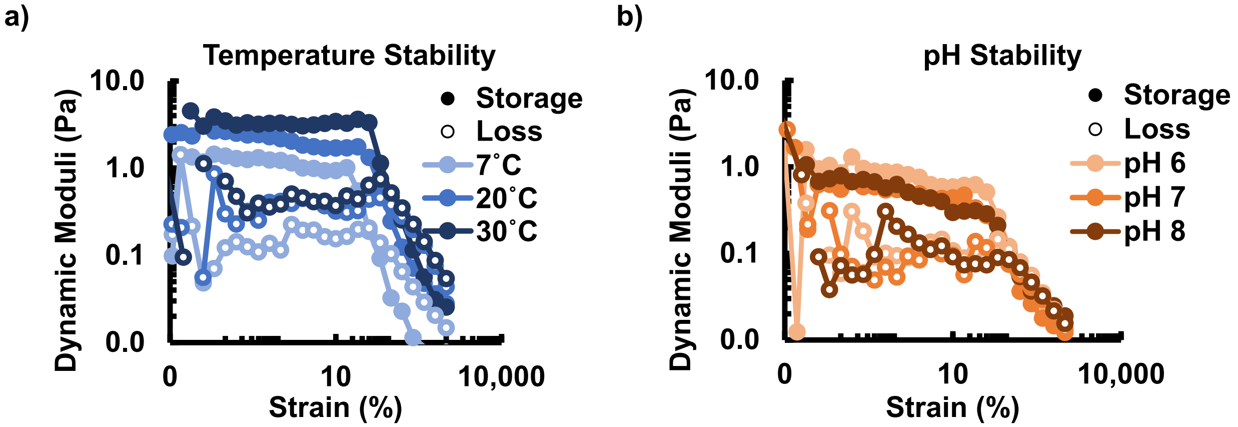

Figure S2: Rheological strain sweeps for a) temperature and b) pH water condition tests conducted at a frequency of 1 rad/s.

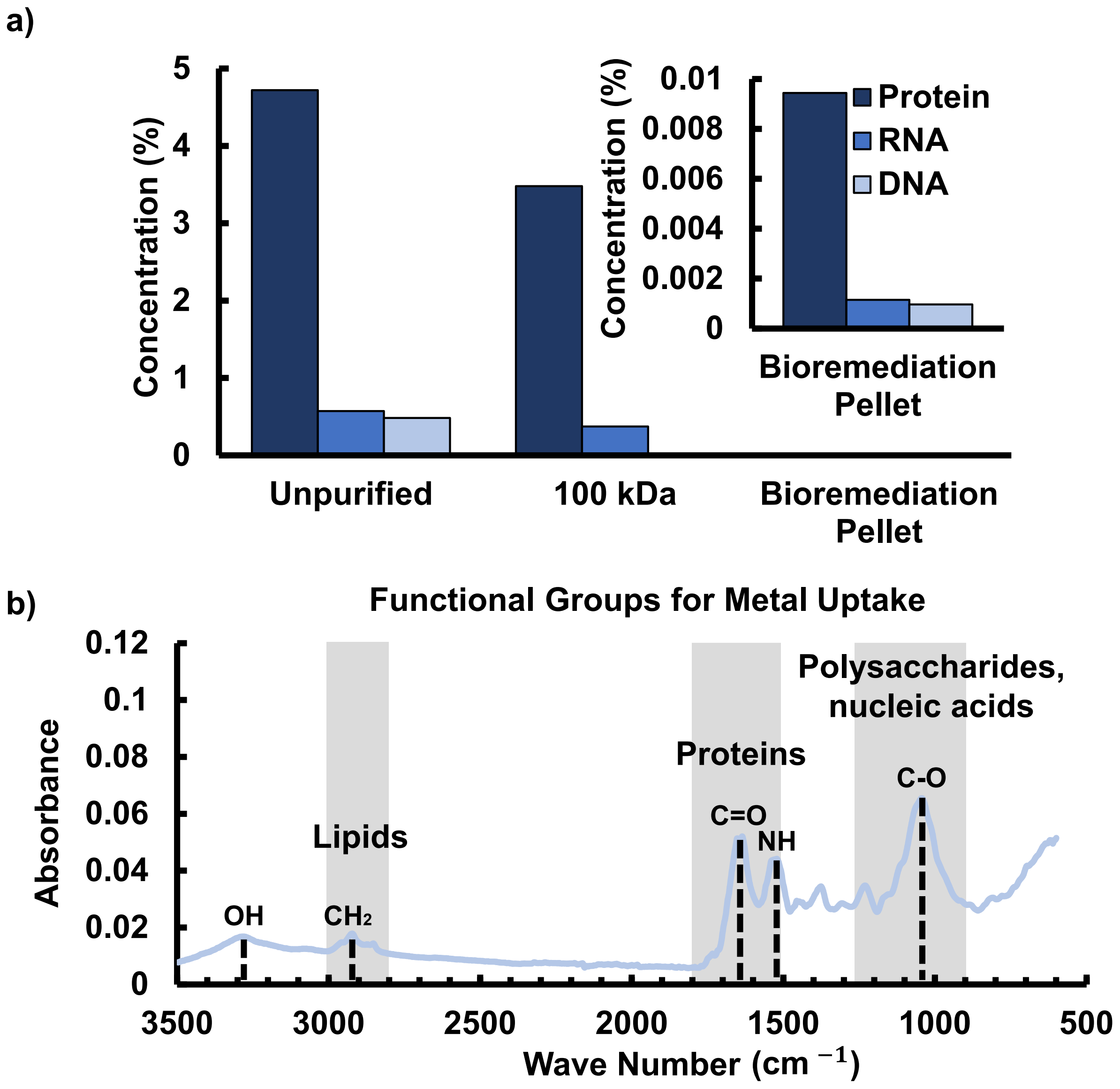

Figure S3: a) Protein, RNA, and DNA composition of *Rheinheimera sp.* T2C2 biofilm measured using a Qubit fluorometer. Inset shows the values for the bioremediation pellet to facilitate visualization. Dialysis results in a loss of low molecular weight small molecules and proteins. b) The biofilm consists of polysaccharides, proteins, and lipids with functional groups that may be suited for heavy metal binding as identified using FTIR-ATR.

2.0 Biosorption of Cobalt using *Rheinheimera sp.* T2C2

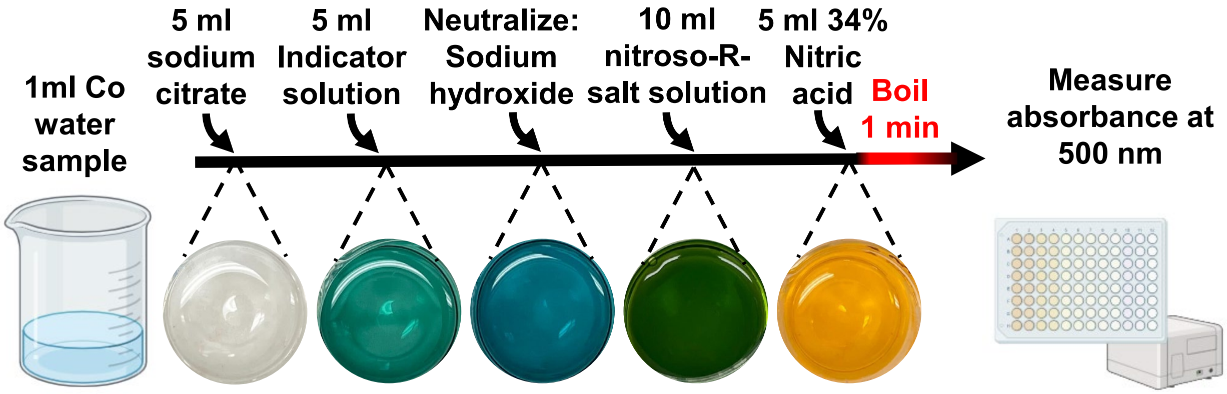

Figure S4: Colorimetric cobalt measurement assay using nitroso-R salt.

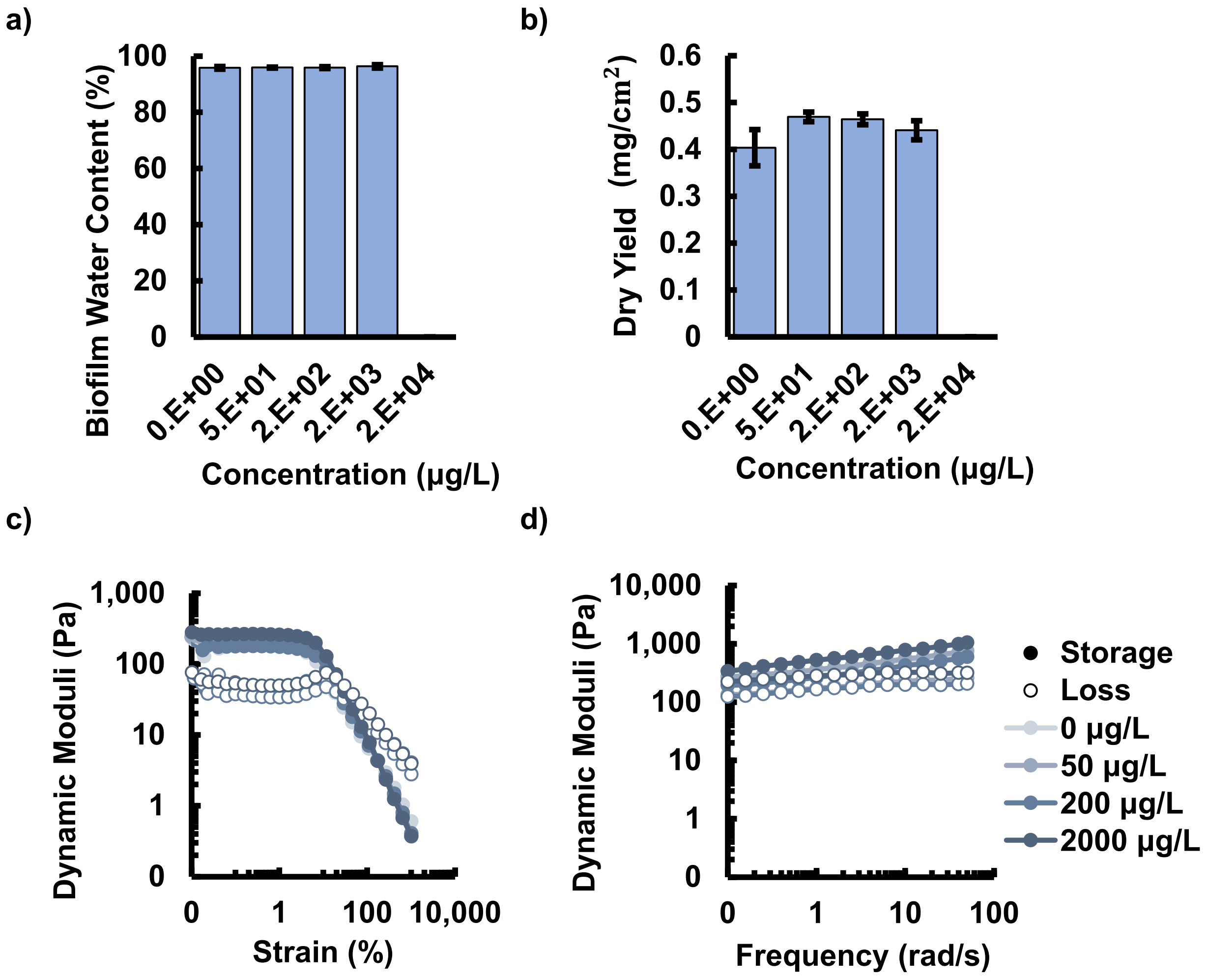

Figure S5: The effect of cobalt concentration for *Rheinheimera sp.* T2C2 biofilm grown on cobalt-LB-agar plates. Cobalt concentrations below 2E+04 μg/ml had minimal effect on a) biofilm water content, Mean values are reported and error bars show standard deviation (n=3). b) Yield and rheological properties as seen in the c) strain sweep at 1 rad/s and d) frequency sweep at 5% strain.

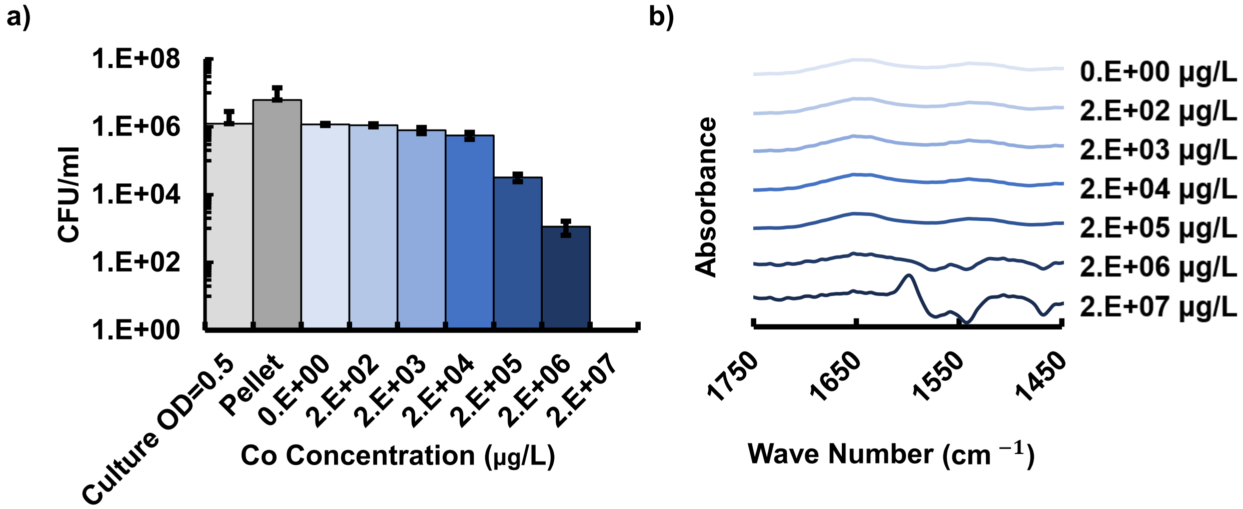

Figure S6: a) Cell viability decreased with increasing metal concentration. Mean values are reported and error bars show standard deviation (n=3). b) Changes in ATR-FTIR spectra were observed for increasing concentration of cobalt.

3.0 The Effect of Varied Water Conditions on Biofilm Biosorption of Cobalt

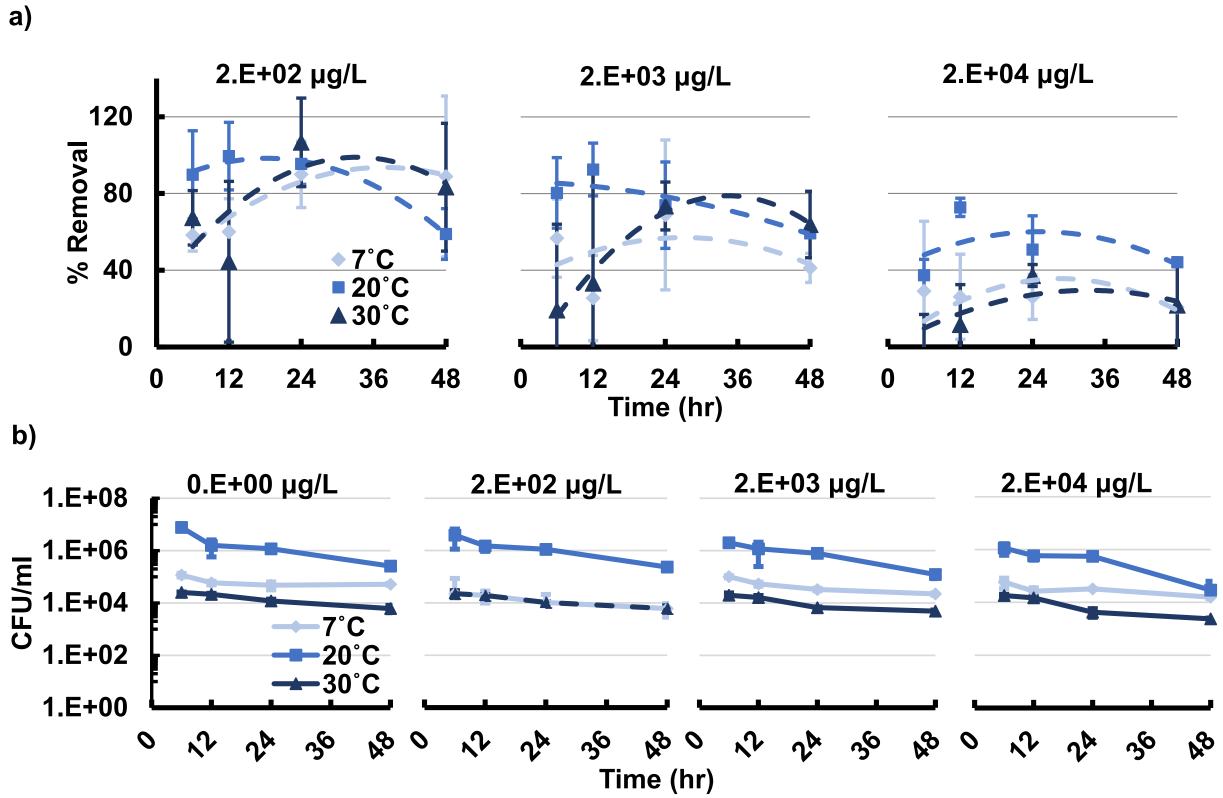

Figure S7: a) The effect of varying water temperature and exposure time on percent cobalt removal showed differing peak uptake times depending on temperature. The percentage metal removed decreased for increasing metal concentration. b) Cell viability in varied water temperatures indicated that T2C2 grows best at a temperature of 20°C. Viability decreases with increased metal exposure time for all temperatures tested. Mean values are reported and error bars show standard deviation (n=3).

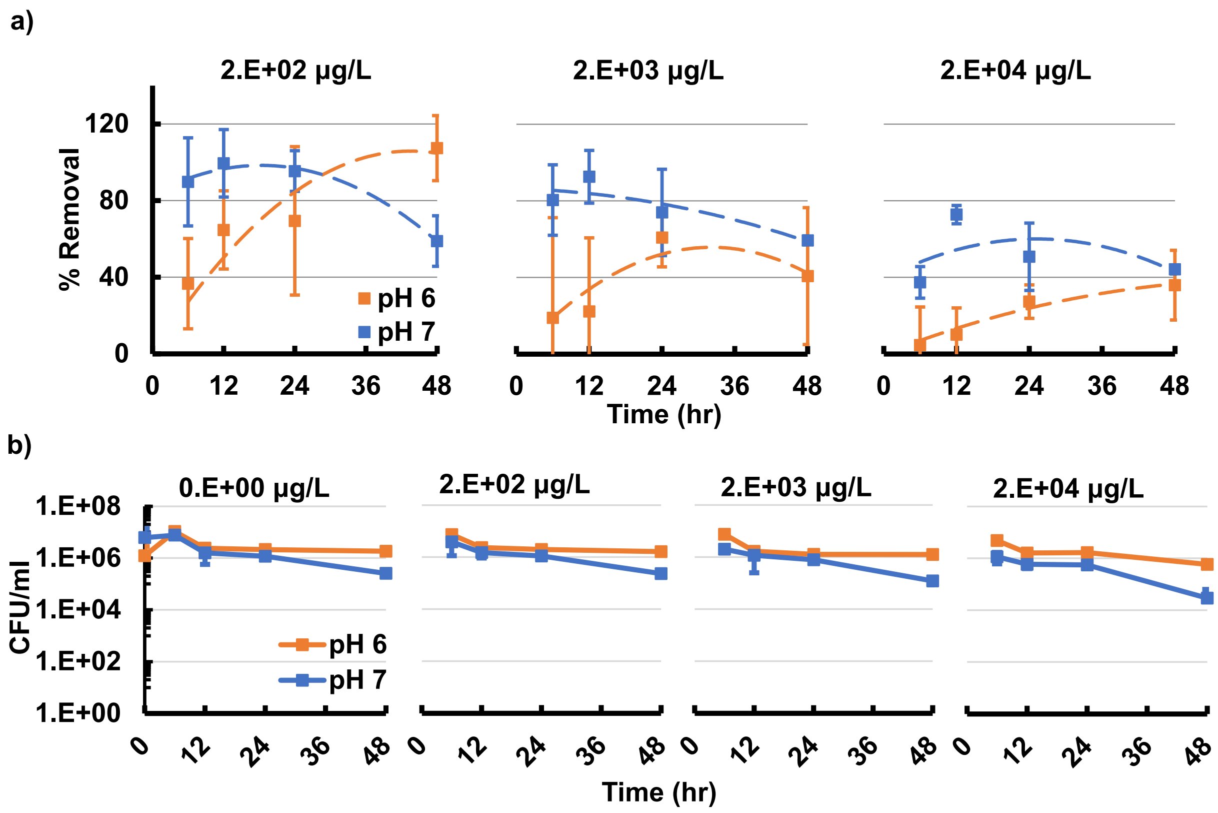

Figure S8: a) Varying water pH and exposure time altered the percent cobalt removal producing different peak uptake times. The percentage metal removed decreased for increasing metal concentration. A higher pH of 7 generally resulted in higher metal uptake that a pH of 6. b) Cell viability for varied water pH indicated similar growth with decreased viability for longer metal exposure times. Mean values are reported and error bars show standard deviation (n=3).

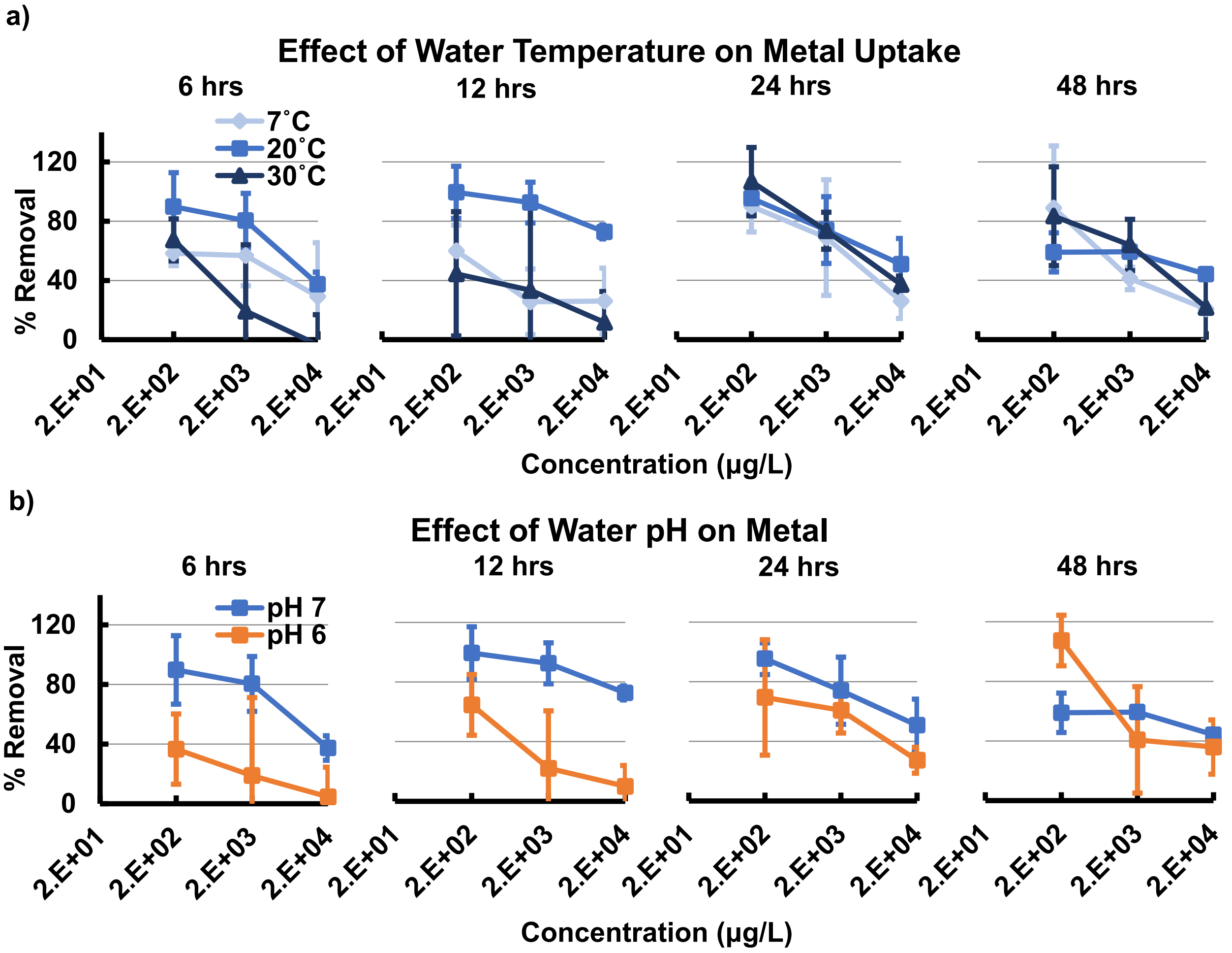

Figure S9: Percent of cobalt removal vs. initial concentration for 6, 12, 24, and 48 hours for a) varied water temperature and b) pH. Mean values are reported and error bars show standard deviation (n=3).

4.0 Biofilm Mode of Metal Uptake

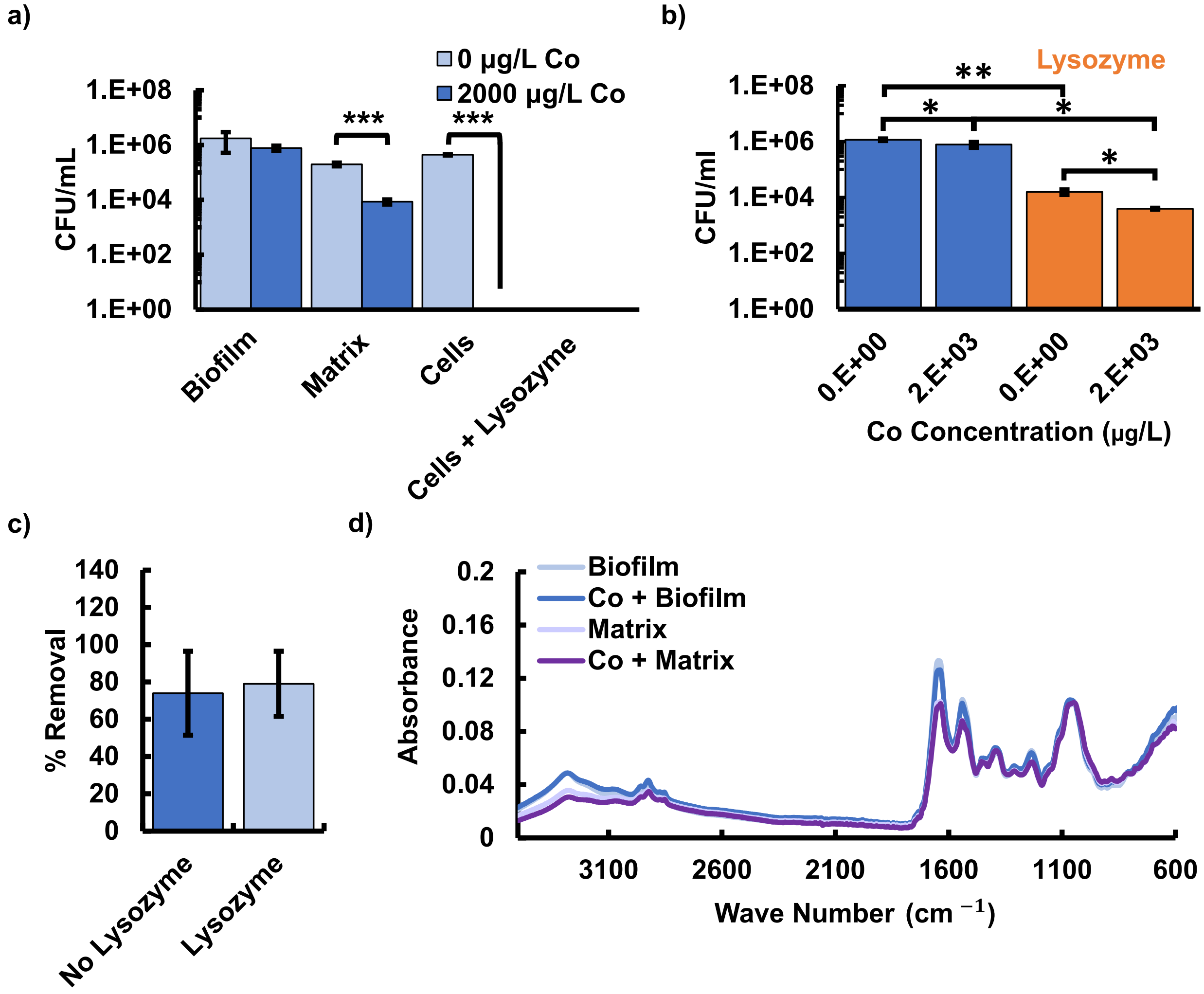

Figure S10: a) Cell viability tests for centrifugation to separate the cells from the biofilm. Note that no cells survived lyophilization of the biofilm. b) Cell viability due to a 6 hr lysozyme treatment, without first separating cells, at a concentration of 5 mg/ml resulted in the majority of cells surviving. c) No statistically significant change in metal removal was observed when cells were treated with lysozyme without initial cell-film separation. Mean values are reported and error bars show standard deviation (n=3), p-values less than 0.05, 0.01, and 0.001 are denoted by *, **, and ***, respectively. d) FTIR results for biofilm with cells and matrix without cells exposed to cobalt concentrations of 0 and 2E+03 μg/L, normalized to carbohydrate peak at 1045 cm^{-1}.

5.0 Cobalt Recovery via Biofilm Protein Degradation

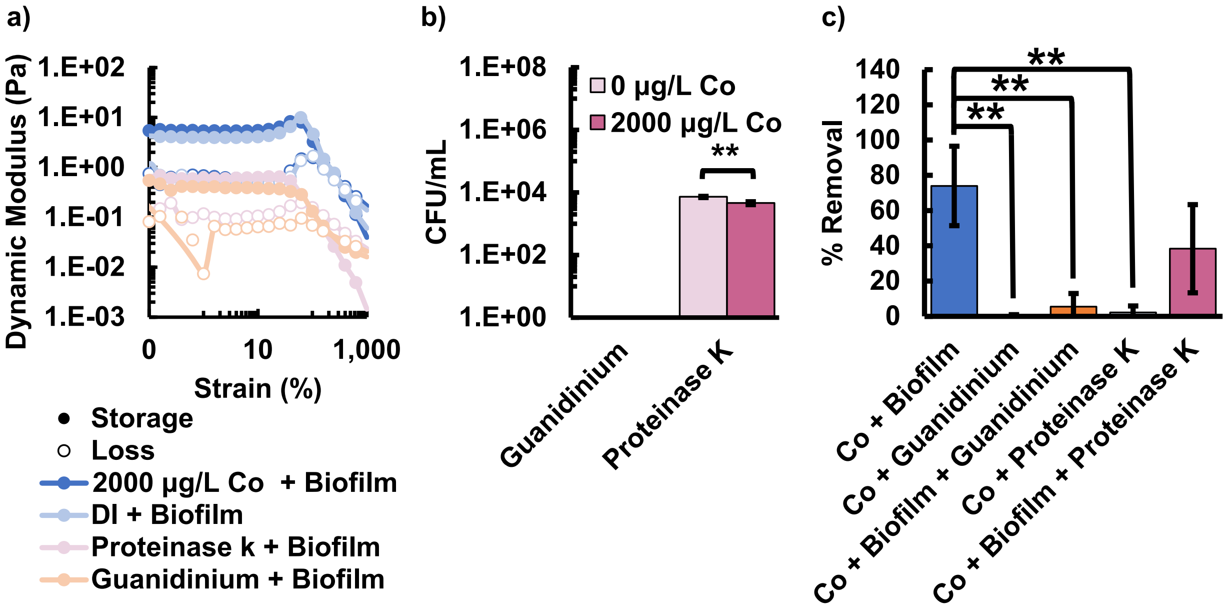

Figure S11: Metal recovery from the biofilm was conducted via protein degradation a) Strain sweep showing biofilm degradation due to proteinase k and guanidinium exposure. b) Cell viability for biofilm degradation using proteinase K (0.2% w/v) and guanidinium iodide (10% w/v) for 6 hours, respectively. c) Percent removal for biofilm and treated groups demonstrating uptake and metal release into free solution. Mean values are reported and error bars show standard deviation (n=3), p-values less than 0.05 and 0.01 are denoted by * and **, respectively.

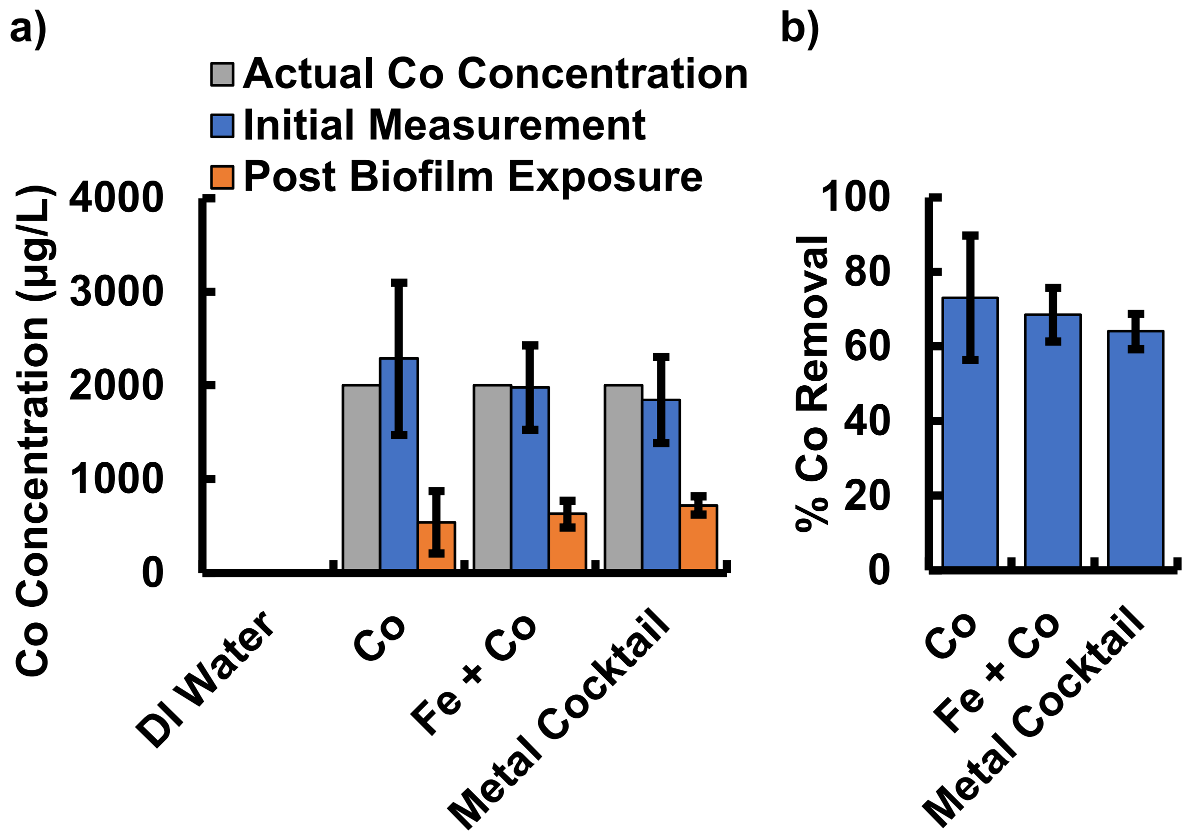

Figure S12: Cobalt uptake and anticipated metal recovery may be altered by the presence of other trace metals in the environment. Solutions of 2000 μg/L cobalt, 2000 μg/L cobalt and 1000 μg/L iron, and a metal cocktail (2000 μg/L cobalt, 1000 μg/L iron, 470 μg/L nickel, 2.2 μg/L manganese, and 120 μg/L zinc) were exposed to biofilm to determine how cobalt uptake would change. a) The cobalt actual known concentrations, initial measurements, and post biofilm exposure concentrations were measured. b) The percent cobalt removed decreases slightly with the presence of additional metals. However, changes are not statistically significant likely due to the low metal concentrations and lack of binding site saturation in the biofilm. Plots report mean values and error bars show standard deviation (n=3).
